# Association of co-occurring opioid or other substance use disorders with increased healthcare utilization in patients with depression

**DOI:** 10.1038/s41398-021-01372-0

**Published:** 2021-05-03

**Authors:** Veer Vekaria, Budhaditya Bose, Sean M. Murphy, Jonathan Avery, George Alexopoulos, Jyotishman Pathak

**Affiliations:** 1 Department of Population Health Sciences, Weill Cornell Medicine, New York, NY USA; 2 Department of Psychiatry, Weill Cornell Medicine, New York, NY USA

**Keywords:** Addiction, Depression

## Abstract

Substance use disorders (SUDs) commonly co-occur with mental illness. However, the ongoing addiction crisis raises the question of how opioid use disorder (OUD) impacts healthcare utilization relative to other SUDs. This study examines the utilization patterns of patients with major depressive disorder (MDD) and: (1) co-occurring OUD (MDD-OUD); (2) a co-occurring SUD other than OUD (MDD-NOUD); and (3) no co-occurring SUD (MDD-NSUD). We analyzed electronic health records (EHRs) derived from multiple health systems across the New York City (NYC) metropolitan area between January 2008 and December 2017. 11,275 patients aged ≥18 years with a gap of 30–180 days between 2 consecutive MDD diagnoses and an antidepressant prescribed 0–180 days after any MDD diagnosis were selected, and prevalence of any SUD was 24%. Individuals were stratified into comparison groups and matched on age, gender, and select underlying comorbidities. Prevalence rates and encounter frequencies were measured and compared across outpatient, inpatient, and emergency department (ED) settings. Our key findings showed that relative to other co-occurring SUDs, OUD was associated with larger increases in the rates and odds of using substance-use-related services in all settings, as well as services that integrate mental health and substance abuse treatments in inpatient and ED settings. OUD was also associated with larger increases in total encounters across all settings. These findings and our proposed policy recommendations could inform efforts towards targeted OUD interventions, particularly for individuals with underlying mental illness whose treatment and recovery are often more challenging.

## Introduction

In 2018, nearly 20% of US adults experienced mental illness, and nearly 20% of those with mental illness experienced a co-occurring substance use disorder (SUD)^1^. That same year, more than 4 million US adults with a SUD experienced a major depressive episode, and well over 500,000 of those specifically had an opioid use disorder (OUD)^1–5^.

Major depressive disorder (MDD) and SUDs have a bidirectional relationship: symptoms of one disorder increase and reinforce the risk of the other, making this patient population particularly challenging to treat. Left untreated, patients with co-occurring disorders typically exhibit poorer health outcomes—including greater depressive symptomatology, more severe functional impairment, poorer recovery rates, increased suicidal ideation and attempt, and higher rates of healthcare utilization—compared to those with a diagnosis of MDD alone^2,6–9^. Furthermore, depression has been documented as a risk factor for misusing opioids (for example, to treat symptoms of insomnia and stress), while co-occurring substance use may compromise adherence to and the mood-stabilizing effects of antidepressant medications^2,10^.

Given the significant overlaps, theories suggest addressing the dual care needs of patients with co-occurring MDD and SUD is vital to improving morbidity and mortality outcomes among this population^5,6,11^. In fact, studies have shown that integrated approaches which coordinate mental health and substance use therapies may produce more effective outcomes than parallel or sequential treatments delivered in separate settings^9,12–17^. For example, such interventions for MDD that simultaneously reduce substance use are more likely to improve symptoms of both disorders, reduce relapse rates, and enhance recovery^18–20^. However, in 2018, only 11% of adults with co-occurring mental illness and SUDs received such integrated treatments, and over 30% received no treatment at all^1^.

On the other hand, patients with MDD or SUD who do ultimately receive treatment are high healthcare utilizers. Primary care patients with MDD typically have more annual healthcare visits, specialist referrals, laboratory tests, and radiologic scans and procedures than patients without MDD^21,22^. In hospitals, depression has been associated with increased risk of inpatient admissions, length of stay, and risk of 30-day readmission^21–24^. Similarly, patients with SUDs typically have increased emergency department (ED) encounters and inpatient hospitalizations^25^. When combined with MDD, SUDs are associated with increased psychiatric and other medical utilization, including hospitalizations, lengths of stay, and costs (largely attributed to increased psychiatric inpatient encounters)^26–29^.

Such trends are especially intriguing when parsing out the various types of SUDs. In particular, the United States is grappling with an epidemic marked by high rates of addiction treatment admissions, hospitalizations, and overdose deaths related to prescription opioids and heroin^5,30,31^. Patients with OUD are particularly costly, as they are the highest healthcare utilizers among patients with SUDs and are more likely to use crisis- and substance use-related services^32–34^. Although OUD shares the same category as other SUDs, several features set OUD apart in terms of specialized management and treatment. For example, withdrawal symptoms from opioids are far more severe than from other substances and can lead to physical dependence in as little as 4–8 weeks^35^. Second, the supply and access to prescription opioids in medical practice fueled the opioid epidemic and provided a key gateway to non-medical heroin use^34,35^. Third, users who relapse on opioids are at a significantly higher risk of overdose and mortality compared to users who relapse on other substances such as cannabis or alcohol^35,36^. Finally, whereas treatment for all other SUDs can take place outside the formal healthcare system, successful treatment of OUD demands patients to be on medications (such as buprenorphine, methadone, or long-acting injectable naltrexone), which in turn requires healthcare providers and clinics to be part of this treatment ‘ecosystem’.

While co-occurring SUDs have been associated with increased healthcare utilization in individuals with MDD, it remains unclear to what extent co-occurring OUD and non-OUD SUDs increase healthcare utilization among individuals with MDD. This study aims to fill this knowledge gap by using a pharmacotherapy-treated sample of adult patients to examine the patterns of healthcare services utilization in three groups of patients with MDD: (1) those with co-occurring OUD (MDD-OUD); (2) those with a co-occurring SUD other than OUD (MDD-NOUD); and (3) those with no co-occurring SUD (MDD-NSUD).

## Subjects and methods

### Data source and study sample

**Fig. 1:**
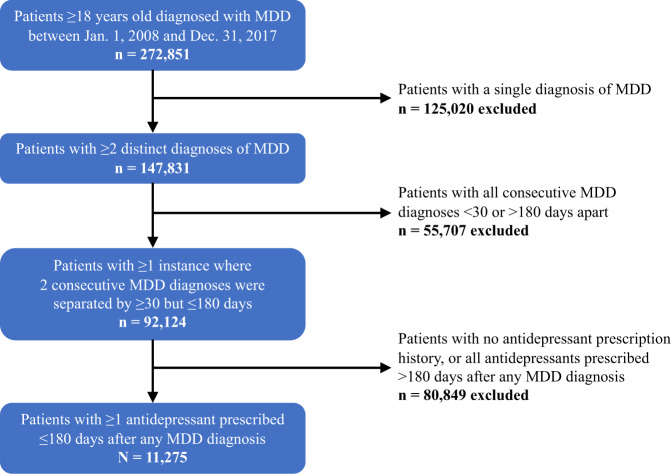
Exclusion cascade used to select the MDD cohort from the INSIGHT CRN dataset. It should be noted that this study examines a niche group of patients with MDD who were treated via pharmacotherapy within a very narrow time window after diagnosis. Because (1) detection standards for depression are not well defined, (2) documentation routines are highly variable particularly for patients with co-occurring SUDs, and (3) overlapping symptomatology makes it difficult for practitioners to deduce a differential diagnosis, we opted to select a highly sensitive case definition that greatly minimizes the inclusion of false positives and yields a sample of highly chronic patients^60–64^.

Using fully de-identified electronic health record (EHR) data from the PCORI-funded INSIGHT Clinical Research Network (CRN), we identified and analyzed the utilization of patients during the decade from January 2008 to December 2017. The INSIGHT CRN compiles EHRs of 12 million patients from six large medical centers across New York City, including Montefiore Medical Center, New York-Presbyterian Hospital, Mount Sinai Health System, New York University Langone Medical Center, Columbia University Medical Center, and Weill Cornell Medicine^37^. Institutional IRB exemption was granted for use of this database for research. Data were extracted from EHRs using the Observational Medical Outcomes Partnership (OMOP) Common Data Model. Figure 1 outlines the predefined inclusion and exclusion criteria used to select the MDD cohort (*n* = 11,275) from the INSIGHT CRN dataset.

**Fig. 2:**
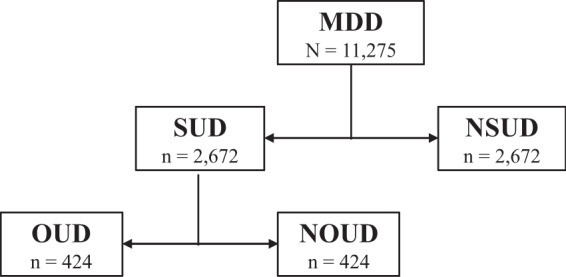
Classification of the MDD-OUD, MDD-NOUD, and MDD-NSUD comparison groups. Nearest neighbor matching was the technique we used for propensity score matching, and this analysis was performed using the MatchIt package in R version 3.6.0. The covariates (age, gender, and comorbidity) were matched using the propensity score distance measure and a one-to-one (1:1 ratio) matching approach was used to select the best control subject for each case subject. The specific comorbidities applied in the propensity scoring algorithm are listed in Table 1 under “Clinical Status,” however note that the overall and subcategories of “Co-Occurring Mental Health Disorder(s)” and “Substance Use Disorder (SUD)” were not applied as these variables serve to distinguish the comparison groups. The time frame used for propensity score matching was 1 January 2008 to 31 December 2017 (the full time period of observation for this study). Detailed results in terms of the propensity score matching analysis are included in the Supplementary.

First, to evaluate the overall demographic makeup of our study sample, all 11,275 patients in the MDD cohort were classified by age, gender, race, and ethnicity. To assess the overall health status of our study sample, the MDD cohort was evaluated for prevalence of select common chronic conditions, co-occurring mental health disorders, and SUDs using ICD-9 and ICD-10 codes. Next, to establish the patterns and measures of utilization associated with MDD alone, i.e. in the absence of co-occurring OUD or other SUDs, the MDD cohort was stratified into two groups using ICD-9 and ICD-10 codes: (1) patients with one or more lifetime co-occurring SUDs (MDD-SUD, *n* = 2672) and (2) patients with no lifetime co-occurring SUD (MDD-NSUD, *n* = 8603). To reduce their potentially confounding effects on service use, MDD-SUD was age, gender, and comorbidity-matched to an equally sized sample of MDD-NSUD (*n* = 2672) using nearest-neighbor propensity score matching^38,39^. To isolate OUD from non-OUD SUDs, MDD-SUD was further stratified into two groups using ICD-9 and ICD-10 codes: (1) patients with lifetime co-occurring OUD (MDD-OUD, *n* = 424) and (2) patients with one or more lifetime co-occurring SUDs other than OUD (MDD-NOUD, *n* = 2248). Similarly, MDD-OUD was age, gender, and comorbidity-matched to an equally sized sample of MDD-NOUD (*n* = 424) using nearest neighbor propensity score matching^38,39^. Overall demographics and health status were evaluated for each of the MDD-OUD, MDD-NOUD, and MDD-NSUD comparison groups, and Fig. 2 illustrates the classification and final sample size of each group.

### Measures and data analysis

First, to understand system-level patterns of utilization, we calculated prevalence rates of healthcare utilization among MDD-OUD, MDD-NOUD, and MDD-NSUD. Differences were compared using chi-squared tests, and *p* < 0.05 was used as the threshold for defining statistical significance. Then, to ascertain between which groups the differences mostly occurred, multiple pairwise comparisons were performed using two-proportions *Z*-tests, and a Bonferroni-corrected *p* < 0.05/3 or 0.0167 was used as the threshold for defining statistical significance. Odds ratios (ORs) with 95% confidence intervals (CIs) were also computed to estimate effect sizes. Second, to understand individual-level patterns of utilization, visit frequencies were measured for each individual who had at least one encounter in MDD-OUD, MDD-NOUD, and MDD-NSUD and median, first quartile, and third quartile values were computed for each group. Multiple pairwise comparisons were performed using Mann–Whitney *U* (Wilcoxon rank-sum) tests to determine between which groups the differences mostly occurred, and a Bonferroni-corrected *p* < 0.05/3 or 0.0167 was used as the threshold for defining statistical significance. The data met the respective assumptions of all statistical tests performed. R version 3.6.0 was used for all analyses^40^.

## Results

### Demographic and clinical characteristics

According to Table 1, our study sample largely consisted of patients aged 45 years and older (79%), female (69%), and non-Hispanic or Latino (56%). The largest race categories represented were White (32%), Black or African American (9%), and Asian (4%). Of note, the race and ethnicity categories represented are reflective of unreported race and ethnicity data in the INSIGHT CRN dataset, however they are generalizable as we have no reason to believe that uncoded data was unevenly distributed across racial and ethnic categories. The most common clinical comorbidities in patients with MDD were hypertension (53%), hyperlipidemia (51%), co-occurring mental health disorders (45%), in particular bipolar disorder (43%), and anemia (35%). In addition, the prevalence of any SUD in patients with MDD was 24%. Maximum prevalence was found with tobacco use disorder (16%), followed by other SUDs (8%) and alcohol use disorder (7%).

**Table 1:** Demographic and clinical characteristics (%) of the 11,275 patients included in this study.

	MDD *n* = 11,275	MDD-OUD *n* = 424	MDD-NOUD *n* = 424	MDD-NSUD *n* = 2672
*Demographics*				
Age				
18–24	2	0	1	3
25–44	19	17	18	21
45–64	33	54	49	32
≥65	46	29	32	43
Gender				
Female	69	56	59	43
Race				
White	32	21	19	30
Black or African American	9	16	17	9
Asian	4	<1	1	4
American Indian or Alaska Native	<1	0	<1	<1
Native Hawaiian or Other Pacific Islander	<1	<1	0	<1
Ethnicity				
Not Hispanic or Latino	56	66	63	56
Hispanic or Latino	13	15	14	14
*Clinical status*				
Hypertension	53	67	70	61
Hyperlipidemia	51	51	52	56
Co-occurring mental health disorder(s)	45	84	69	46
Bipolar disorder	43	83	67	44
Psychosis	13	23	14	6
Personality disorders	5	25	14	3
Schizoaffective disorder	<1	<1	0	<1
Anemia	35	62	64	44
Diabetes	26	42	42	33
Rheumatoid arthritis, osteoarthritis	24	37	39	29
Substance use disorder (SUD)	24	60	26	0
Tobacco use disorder	16	65	71	0
Other SUD	8	33	18	0
Alcohol use disorder	7	30	38	0
Cannabis use disorder	4	27	23	0
Stimulant use disorder	4	5	2	0
Opioid use disorder	4	100	0	0
Chronic kidney disease	22	42	42	30
Ischemic heart disease	21	30	29	27
Asthma	21	48	47	30
Cataract	19	20	20	21
Obesity	17	29	30	23
Acquired hypothyroidism	16	17	16	15
COPD	15	36	38	25
Osteoporosis	15	13	13	14
Alzheimer’s disease	14	8	10	12
Glaucoma	11	11	10	12
Stroke, transient ischemic attack	10	14	18	12
Peripheral vascular disease	10	20	19	13
Atrial fibrillation	9	10	10	9
Benign prostatic hyperplasia	6	6	6	7

Demographic variables included age (18–24, 25–44, 45–64, or ≥65); gender; race (White, Black/African American, Asian, American Indian/Alaska Native, or Native Hawaiian/other Pacific Islander); and ethnicity (Hispanic/Latino or not Hispanic/Latino). Using the Chronic Conditions Data Warehouse (CCW) diagnostic criteria available from the Centers for Medicare and Medicaid Services (CMS), common underlying conditions potentially related to service use included acquired hypothyroidism; Alzheimer’s disease; anemia; asthma; atrial fibrillation; benign prostatic hyperplasia; cataract; chronic kidney disease; chronic obstructive pulmonary disease (COPD); diabetes; glaucoma; hyperlipidemia; hypertension; ischemic heart disease; obesity; osteoporosis; peripheral vascular disease; rheumatoid arthritis/osteoarthritis; and stroke/transient ischemic attack^65^. Co-occurring mental health disorders (bipolar disorder, psychosis, schizoaffective disorder, and personality disorders (paranoid, schizoid, antisocial, borderline, histrionic, obsessive, avoidant, dependent, narcissistic, and other)), and SUDs (alcohol, cannabis, opioid, stimulant, tobacco, and other) were also evaluated. The prevalence data for the overall “Co-Occurring Mental Health Disorder(s)” and overall “Substance Use Disorder (SUD)” categories represent the percentage of patients with a history of one or more of the listed specific subcategories.

**Table 2:** A: Prevalence of any service use (%) in MDD-OUD, MDD-NOUD, and MDD-NSUD, 2008–2017. B: Number of encounters among patients who used services (median (Q1-Q3)) in MDD-OUD, MDD-NOUD, and MDD-NSUD, 2008–2017.

Care setting	Encounter type	MDD-OUD	MDD-NOUD	MDD-NSUD	*P*_*χ*2_	MDD-OUD vs. MDD-NOUD		MDD-OUD vs. MDD-NSUD		MDD-NOUD vs. MDD-NSUD		Care setting	Encounter type	MDD-OUD	MDD-NOUD	MDD-NSUD	MDD-OUD vs. MDD-NOUD	MDD-OUD vs. MDD-NSUD	MDD-NOUD vs. MDD-NSUD
						*P*_*Z*_	OR (95% CI)	*P*_*Z*_	OR (95% CI)	*P*_Z_	OR (95% CI)						*P*_U_	*P*_U_	*P*_U_
Outpatient	Any type	96	98	99	*p* < 0.0001	*p* = 0.2047	0.53 (0.21–1.24)	*p* < 0.0001	0.21 (0.10–0.41)	*p* = 0.0430	0.39 (0.17–0.95)	Outpatient	Any type	89 (32–185)	68 (29–151)	44 (19–100)	*p* = 0.0027	*p* < 0.0001	*p* < 0.0001
	Psychiatric	80	83	92	*p* < 0.0001	*p* = 0.2158	0.79 (0.56–1.12)	*p* < 0.0001	0.33 (0.25–0.44)	*p* < 0.0001	0.42 (0.32–0.57)		Psychiatric	11 (4–28)	9 (4–18)	8 (4–16)	*p* = 0.0199	*p* = 0.0003	*p* = 0.5565
	Substance use-related	64	51	<1	*p* < 0.0001	*p* = 0.0001	1.72 (1.31–2.27)	*p* < 0.0001	771 (368–1928)	*p* < 0.0001	448 (214–1188)		Substance use-related	6 (2–20)	3 (1–6)	2 (1–2)	*p* < 0.0001	*p* = 0.0068	*p* = 0.0263
	Integrated	60	55	<1	*p* < 0.0001	*p* = 0.1446	1.24 (0.94–1.63)	*p* < 0.0001	180 (116–294)	*p* < 0.0001	145 (94–237)		Integrated	5 (2–13)	3 (1–7)	2 (1–6)	*p* < 0.0001	*p* = 0.0082	*p* = 0.2277
	Other	91	94	95	*p* = 0.0067	*p* = 0.0910	0.62 (0.36–1.04)	*p* = 0.0022	0.55 (0.39–0.81)	*p* = 0.7062	0.89 (0.59–1.41)		Other	47 (16–113)	45 (17–101)	31 (11–73)	*p* = 0.6332	*p* < 0.0001	*p* < 0.0001
Inpatient	Any type	81	74	46	*p* < 0.0001	*p* = 0.0326	1.44 (1.04–2.00)	*p* < 0.0001	4.91 (3.83–6.36)	*p* < 0.0001	3.40 (2.71–4.30)	Inpatient	Any type	8 (3–17)	5 (3–10)	3 (2–7)	*p* < 0.0001	*p* < 0.0001	*p* < 0.0001
	Psychiatric	44	48	36	*p* < 0.0001	*p* = 0.3704	0.88 (0.67–1.15)	*p* = 0.0022	1.39 (1.13–1.71)	*p* < 0.0001	1.59 (1.29–1.95)		Psychiatric	4 (2–9)	3 (1–5)	3 (1–5)	*p* = 0.0003	*p* < 0.0001	*p* = 0.3689
	Substance Use-Related	37	28	<1	*p* < 0.0001	*p* = 0.0102	1.48 (1.11–1.97)	*p* < 0.0001	712 (227–4987)	*p* < 0.0001	483 (153–3051)		Substance use-related	2 (1–4)	1 (1–2)	1 (1–1)	*p* < 0.0001	*p* = 0.1272	*p* = 0.2949
	Integrated	72	57	<1	*p* < 0.0001	*p* < 0.0001	1.92 (1.45–2.56)	*p* < 0.0001	509 (295–980)	*p* < 0.0001	265 (155–490)		Integrated	4 (2–7)	2 (1–4)	1 (1–1)	*p* < 0.0001	*p* < 0.0001	*p* = 0.0002
	Other	34	36	30	*p* = 0.0166	*p* = 0.7191	0.94 (0.71–1.25)	*p* = .0729	1.23 (0.99–1.52)	*p* = 0.0175	1.31 (1.05–1.62)		Other	2 (1–5)	2 (1–5)	2 (1–4)	*p* = 0.8417	*p* = 0.0338	*p* = 0.0057
ED	Any Type	84	82	52	*p* < 0.0001	*p* = 0.4656	1.16 (0.81–1.66)	*p* < 0.0001	4.88 (3.75–6.45)	*p* < 0.0001	4.20 (3.27–5.48)	ED	Any Type	13 (6–30)	8 (4–17)	5 (2–11)	*p* < 0.0001	*p* < 0.0001	*p* < 0.0001
	Psychiatric	25	22	13	*p* < 0.0001	*p* = 0.2583	1.22 (0.89–1.67)	*p* < 0.0001	2.25 (1.75–2.86)	*p* < 0.0001	1.85 (1.42–2.38)		Psychiatric	1 (1–3)	1 (1–2)	1 (1–2)	*p* = 0.3374	*p* = 0.0657	*p* = 0.6101
	Substance Use-Related	26	15	<1	*p* < 0.0001	*p* < 0.0001	2.03 (1.44–2.88)	*p* < 0.0001	825 (187–16384)	*p* < 0.0001	407 (91–9395)		Substance use-related	1 (1–3)	1 (1–2)	1 (1–1)	*p* = 0.0228	*p* = 0.3758	*p* = 0.5156
	Integrated	24	16	<1	*p* < 0.0001	*p* = 0.0062	1.63 (1.16–2.30)	*p* < 0.0001	267 (100–1105)	*p* < 0.0001	164 (61–688)		Integrated	1 (1–3)	1 (1–2)	1 (1–1)	*p* = 0.1043	*p* = 0.1520	*p* = 0.2013
	Other	73	72	43	*p* < 0.0001	*p* = 0.8177	1.05 (0.78–1.42)	*p* < 0.0001	3.57 (2.85–4.50)	*p* < 0.0001	3.41 (2.73–4.28)		Other	6 (3–15)	4 (2–9)	3 (1–7)	*p* = 0.0007	*p* < 0.0001	*p* = 0.0002

To measure healthcare services use, visits were classified into three care settings: outpatient, inpatient, and ED. To better understand the nature and relevance of these visits, we defined and classified them into four encounter types based on encounter diagnosis: psychiatric only; substance use-related only; integrated; and other. “Psychiatric only” encounters included episodes of care with ICD-9 and ICD-10 codes for depression, bipolar disorder, psychosis, schizoaffective disorder, and personality disorders (paranoid, schizoid, antisocial, borderline, histrionic, obsessive, avoidant, dependent, narcissistic, and other). “Substance use-related only” encounters included episodes of care with ICD-9 and ICD-10 codes related to alcohol, opioids, cannabis, sedatives/hypnotics/anxiolytics, cocaine, stimulants, hallucinogens, nicotine, inhalants, and other substances. “Psychiatric only” excluded episodes of care that met our inclusion criteria for “substance use-related only,” and vice versa. However, episodes of care that met our inclusion criteria for both “psychiatric only” and “substance use-related only” were classified as “integrated” encounters. Finally, episodes of care that met none of the criteria for “psychiatric only,” “substance use-related only,” or “integrated” were classified as “other” encounters. In Table 2A, a *p* < 0.05 was used as the threshold of significance in the chi-squared tests, and to control for type I error, a Bonferroni-corrected *p* < 0.05/3 or 0.0167 was used as the threshold of significance in the two-proportions *Z*-tests for the multiple pairwise comparisons. In Table 2B, a Bonferroni-corrected *p* < 0.05/3 or 0.0167 was used as the threshold of significance in the Mann–Whitney *U* (Wilcoxon rank-sum) tests for the multiple pairwise comparisons. The *p* values are based on bivariate analyses, and additional safeguards (including restrictive inclusion/exclusion criteria and propensity score matching) were implemented in other parts of the method to reduce the influence of relevant factors.

### Increased prevalence of healthcare services use

#### Outpatient

Among patients with MDD, both co-occurring OUD and non-OUD SUDs were associated with increased rates of using substance use-related outpatient services (*p* < 0.001), but OUD was associated with a larger increase (*p* < 0.001, OR = 1.72, 95% CI = 1.31–2.27). MDD-OUD used substance use-related outpatient services the most (64%) compared to MDD-NOUD (51%) and MDD-NSUD (<1%). Also, OUD and non-OUD SUDs were associated with increased rates of using integrated outpatient services (*p* < 0.001). MDD-NSUD used integrated outpatient services the least (<1%) compared to MDD-NOUD (55%) and MDD-OUD (60%).

#### Inpatient

Among patients with MDD, co-occurring OUD and non-OUD SUDs were associated with increased rates of inpatient health services use (*p* < 0.001). MDD-NSUD used inpatient services the least (46%) compared to MDD-NOUD (74%) and MDD-OUD (81%). Further analysis of inpatient services stratified by service type showed that OUD and non-OUD SUDs were associated with increased rates of using psychiatric inpatient services (MDD-OUD *p* = 0.0022; MDD-NOUD *p* < 0.001). MDD-NOUD used psychiatric inpatient services the most (48%), followed by MDD-OUD (44%) and MDD-NSUD (36%). Also, both OUD and non-OUD SUDs were associated with increased rates of using substance use-related inpatient services (*p* < 0.001), but OUD was associated with a larger increase (*p* = 0.0102, OR = 1.48, 95% CI = 1.11–1.97). MDD-OUD used substance use-related inpatient services the most (37%) compared to MDD-NOUD (28%) and MDD-NSUD (<1%). Finally, both OUD and non-OUD SUDs were associated with increased rates of using integrated inpatient services (*p* < 0.001), but OUD was associated with a larger increase (*p* < 0.001, OR = 1.92, 95% CI = 1.45–2.56). MDD-OUD used integrated inpatient services the most (72%), followed by MDD-NOUD (57%) and MDD-NSUD (<1%).

#### ED

Among patients with MDD, co-occurring OUD and non-OUD SUDs were associated with increased rates of ED health services use (*p* < 0.001). MDD-NSUD used ED services the least (52%) compared to MDD-NOUD (82%) and MDD-OUD (84%). Further analysis of ED services stratified by the service nature showed that OUD and non-OUD SUDs were associated with increased rates of using psychiatric ED services (*p* < 0.001). MDD-NSUD used psychiatric ED services the least (13%) compared to MDD-NOUD (22%) and MDD-OUD (25%). Also, both OUD and non-OUD SUDs were associated with increased rates of using substance use-related ED services (*p* < 0.001), but OUD was associated with a larger increase (*p* < 0.001, OR = 2.03, 95% CI = 1.44–2.88). MDD-OUD used substance use-related ED services the most (26%), followed by MDD-NOUD (15%) and MDD-NSUD (<1%). Furthermore, both OUD and non-OUD SUDs were associated with increased rates of using integrated ED services (*p* < 0.001), but OUD was associated with a larger increase (*p* = 0.0062, OR = 1.63, 95% CI = 1.16–2.30). MDD-OUD used integrated ED services the most (24%) compared to MDD-NOUD (16%) and MDD-NSUD (<1%). Finally, OUD and non-OUD SUDs were associated with increased rates of using other medical ED services (*p* < 0.001). MDD-NSUD used other medical ED services the least (43%) compared to MDD-NOUD (72%) and MDD-OUD (73%).

### Increased encounters among users of healthcare services

#### Outpatient

Among MDD patients who used any outpatient services, both OUD and non-OUD SUDs were associated with increased outpatient encounters (*p* < 0.001), but OUD was associated with a larger increase (*p* = 0.0027). On average, MDD-OUD had more outpatient visits (89) than MDD-NOUD (68) and MDD-NSUD (44). Further analysis of outpatient visits stratified by the visit type showed that only OUD was associated with increased psychiatric outpatient encounters (*p* < 0.001). On average, MDD-OUD had more psychiatric outpatient visits (11) than MDD-NOUD (9) and MDD-NSUD (8). Also, only OUD was associated with increased substance use-related outpatient encounters (*p* = 0.0068). On average, MDD-OUD had more substance use-related outpatient visits (6) than MDD-NOUD (3) and MDD-NSUD (2). Furthermore, only OUD was associated with increased integrated outpatient encounters (*p* = 0.0082). On average, MDD-OUD had more integrated outpatient visits (5) than MDD-NOUD (3) and MDD-NSUD (2). Finally, OUD and non-OUD SUDs were associated with increased other medical outpatient encounters (*p* < 0.001). On average, MDD-NSUD had fewer other medical outpatient visits (31) than MDD-NOUD (45) and MDD-OUD (47).

#### Inpatient

Among MDD patients who used any inpatient services, both OUD and non-OUD SUDs were associated with increased inpatient encounters (*p* < 0.001), but OUD was associated with a larger increase (*p* < 0.001). On average, MDD-OUD had more inpatient visits (8) than MDD-NOUD (5) and MDD-NSUD (3). Further analysis of inpatient visits stratified by the visit type showed that only OUD was associated with increased psychiatric inpatient encounters (*p* < 0.001). On average, MDD-OUD had more psychiatric inpatient visits (4) than MDD-NOUD (3) and MDD-NSUD (3). Furthermore, both OUD and non-OUD SUDs were associated with increased integrated inpatient encounters (*p* < 0.001), but OUD was associated with a larger increase (*p* < 0.001). On average, MDD-OUD had more integrated inpatient visits (4) than MDD-NOUD (2) and MDD-NSUD (1).

#### ED

Among MDD patients who used any ED services, both OUD and non-OUD SUDs were associated with increased ED encounters (*p* < 0.001), but OUD was associated with a larger increase (*p* < 0.001). On average, MDD-OUD had more ED visits (13) than MDD-NOUD (8) and MDD-NSUD (5). Further analysis of ED visits stratified by the visit type showed that both OUD and non-OUD SUDs were associated with increased other medical ED encounters (*p* < 0.001), but OUD was associated with a larger increase (*p* < 0.001). On average, MDD-OUD had more other medical ED visits (6) than MDD-NOUD (4) and MDD-NSUD (3).

## Discussion

Behavioral features and clinical needs that distinguish OUD from other SUDs reflect in the differential utilization patterns we observed. For example, our finding that OUD was associated with larger increases in total encounters, particularly the rates and odds of using substance use-related and integrated services, in both inpatient and ED settings is consistent with current literature and the significantly greater risks of life-threatening overdose and mortality associated with OUD compared to other SUDs^32,41–46^.

Conversely, the differential utilization patterns we observed expand on recent findings that could suggest OUD may more strongly reinforce the risk and symptoms of mental illness compared to other SUDs. While little is currently known about OUD in relation to other SUDs among patients with major depression, a recent analysis using a national sample of people with severe mental illness found that those with a co-occurring heroin use disorder were 19 times more likely than those without co-occurring SUDs to have criminal justice system involvement, while those with all other co-occurring SUDs (i.e., apart from OUDs) were only five times more likely. In addition, those with a co-occurring prescription painkiller use disorder were 2.4 times more likely to attempt suicide than those without co-occurring SUDs, while those with all other co-occurring SUDs were 1.8 times more likely^47^. The potential implication that OUD may more strongly reinforce mental illness would lend a new dimension into what is currently understood about the broader bidirectional relationship, and may be further supported by our findings that (1) compared to patients with other SUDs, patients with OUD were more likely to use inpatient and ED services that integrate mental health and substance abuse treatments, and (2) only OUD was associated with increased psychiatric and integrated encounters in both outpatient and longer term inpatient settings.

Current literature suggests that the etiologies and risk factors for mental health disorders and OUD overlap, and that if either is untreated, both will impact patient outcomes. It is important to note that nearly half of the MDD patients included in this study had other co-occurring mental health disorders (45%), and in particular bipolar disorder (43%). In one study, individuals with bipolar depression had more psychiatric hospitalization, mental health-related outpatient visits, social services visits, and ED visits than individuals with unipolar depression^48^. This underscores the complexity of patients presenting to clinics with multiple co-occurring mental illnesses, and moreover sheds light on the overlapping symptomatologies that cloud differential diagnosis in this population^49–51^.

Our analysis of MDD patients indicates that co-occurring OUD and non-OUD SUDs differentially increase utilization across settings, requiring equally differential treatment planning. Whereas the clinical features and needs that distinguish OUD from other SUDs are helpful for understanding treatment of an individual patient, the utilization patterns observed in this study may help guide selection of trade-offs in policy and other interventions. For example, practitioners should screen for opioid dependence in patients who present with mental illness, especially those with a history of substance use including prescription opioids. In addition, practitioners should initiate treatment for OUD prior to evaluating and treating mental health disorders soon thereafter. This is based on an earlier suggestion that co-occurring OUD may more strongly reinforce the risk and symptoms of mental illness compared to other co-occurring SUDs. Further investigation is needed to ascertain whether temporality of mental health and substance abuse treatment impacts long-term outcomes.

Furthermore, among MDD patients with co-occurring OUD, the inpatient utilization rate of integrated services was much higher (72%) as compared to non-integrated psychiatric (44%) and substance use-related (37%) treatment services. Conversely, the outpatient utilization rate of integrated services was the lowest (60%) as compared to non-integrated psychiatric (80%) and substance use-related (64%) treatment services. Our broader inpatient and outpatient results resemble those of a national sample of individuals treated for OUD during a similar study period^52^. Limiting the use of parallel treatments in outpatient settings and instead shifting towards integrated treatment models could be more cost-effective and reduce the rate of medical and psychiatric hospitalizations related to a dual diagnosis^53–55^. For cases that necessitate hospitalization, we agree with the authors of a recent study of SUD readmissions in NYC hospitals who suggested that hospital settings could be useful venues for substance use-related interventions, and could benefit from close coordination with outpatient providers and more targeted discharge planning^56^.

Interestingly, both OUD and non-OUD SUDs were associated with decreased rates of using psychiatric outpatient services in patients with MDD (*p* < 0.001), although supplemental findings suggest these decreases were recaptured by increased integrated outpatient utilization. A potential future research question could examine whether introducing mental health treatment during the treatment for SUDs improves non-integrated outpatient mental health treatment entry and ambulatory care utilization after hospital discharge in dual diagnosis patients.

The findings from this study should be understood within the context of a few methodological limitations associated with the use of EHR data. First, although it had minimal impact on depression, it is important to consider how the transition from ICD-9-CM to ICD-10-CM impacted coding of mental health conditions^57^. Second, the INSIGHT CRN dataset used in this study is inherently biased because it only includes patients who have a MDD diagnosis and/or an antidepressant prescription. Restricting the study cohort to the intersection of depressed patients treated via pharmacotherapy limits our ability to account for patients who receive only non-pharmacological treatments such as psychotherapy and cognitive behavioral therapy (CBT)^58^. Finally, the INSIGHT CRN pertains to health centers within the NYC metropolitan area, limiting our ability to accurately control for prescription drug use patterns (in particular prescription opioids) and generalize findings to a national scale. It is also possible that depressed patients captured within, but prescribed antidepressants outside of, the INSIGHT CRN were excluded from the study cohort.

## Conclusion

The United States continues to make strides in improving access to mental health services^59^. However, the alarmingly high rate of co-occurring substance use among mentally ill patients makes it important to understand how this impacts relevant service utilization and health outcomes. Our key findings showed that relative to other co-occurring SUDs, OUD was associated with larger increases in the rates and odds of using substance-use-related services in all settings, as well as services that integrate mental health and substance abuse treatments in inpatient and ED settings. OUD was also associated with larger increases in total encounters across all settings. Our analysis expands and motivates further inquiry on recent preliminary evidence that could suggest co-occurring OUD may more strongly reinforce the risk and symptoms of mental illness relative to other co-occurring SUDs. We propose several policy recommendations for better managing co-occurring OUD, including prioritized screening and initiation of treatment for opioid dependence, as well as closer coordination between inpatient, ED, and outpatient care settings. In the midst of an ongoing addiction crisis, recognition among stakeholders (i.e., providers, administrators, and policymakers) of the heterogeneity among SUDs will inform targeted, evidence-based interventions for OUD. Further investigation is needed to assess the efficacy of our proposed policy recommendations on long-term health outcomes related to the management of co-occurring OUD.

## Supplementary information

Supplementary

## Data Availability

The data of this study are not publicly available due to privacy and ethical restrictions. Data to support the findings of this study are available upon reasonable request.

## Code availability

The computer code used for the cohort selection and utilization analysis is available upon reasonable request.
