# Supplementary material for: Association of co-occurring opioid or other substance use disorders with increased healthcare utilization in patients with depression
Source: Transl Psychiatry. 2021 May 3;11:265. doi: 10.1038/s41398-021-01372-0 (PMC8093211; doi:10.1038/s41398-021-01372-0)

**SUPPLEMENTARY**

1. **Propensity Score Matching Analysis:**

**MDD-SUD (n = 2 672) to MDD-NSUD (n = 8 603** 🡪 **n = 2 672)**

- 1. Percent Balance Improvement


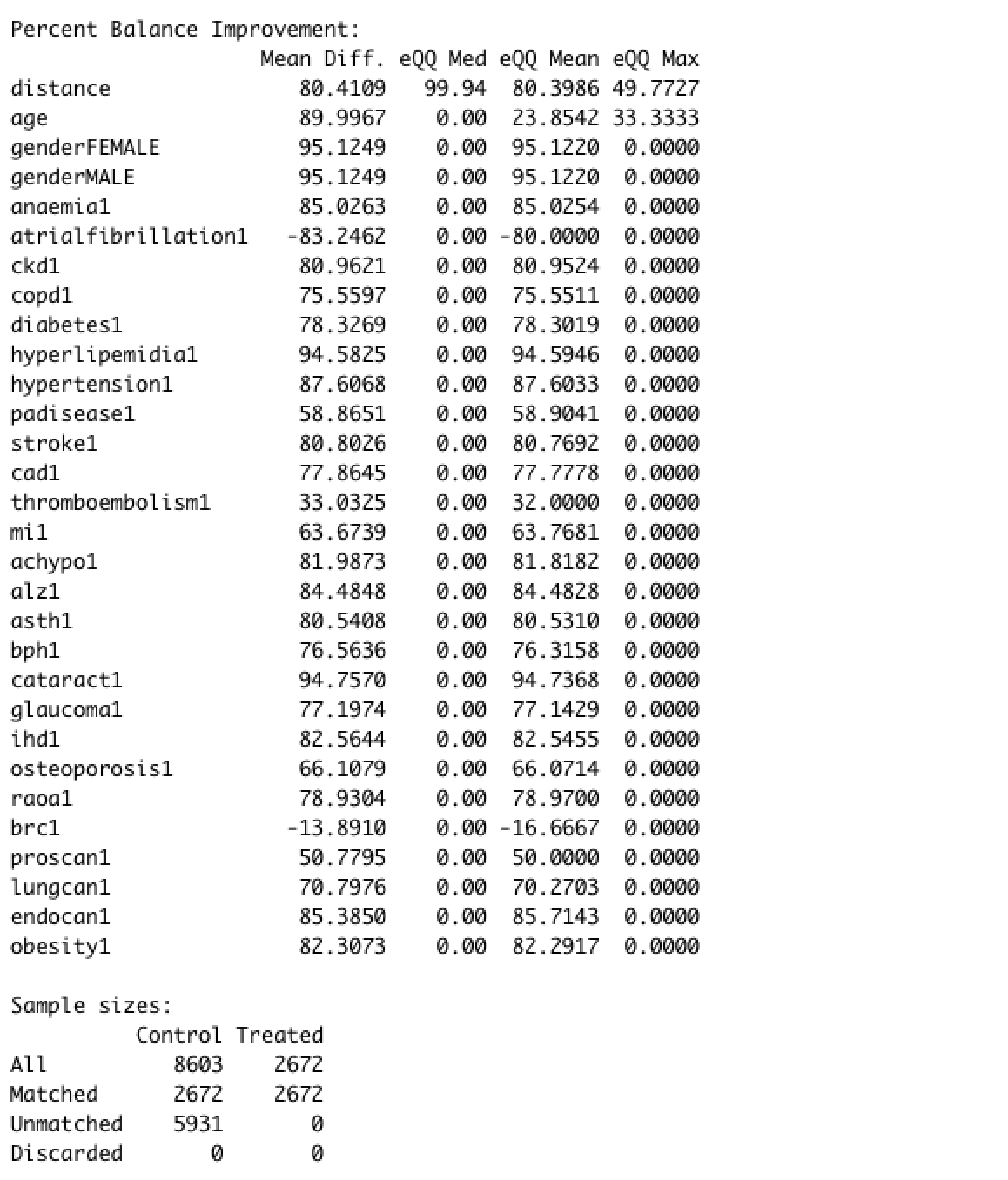


- 1. Distribution of Propensity Scores (Jitter Plot)


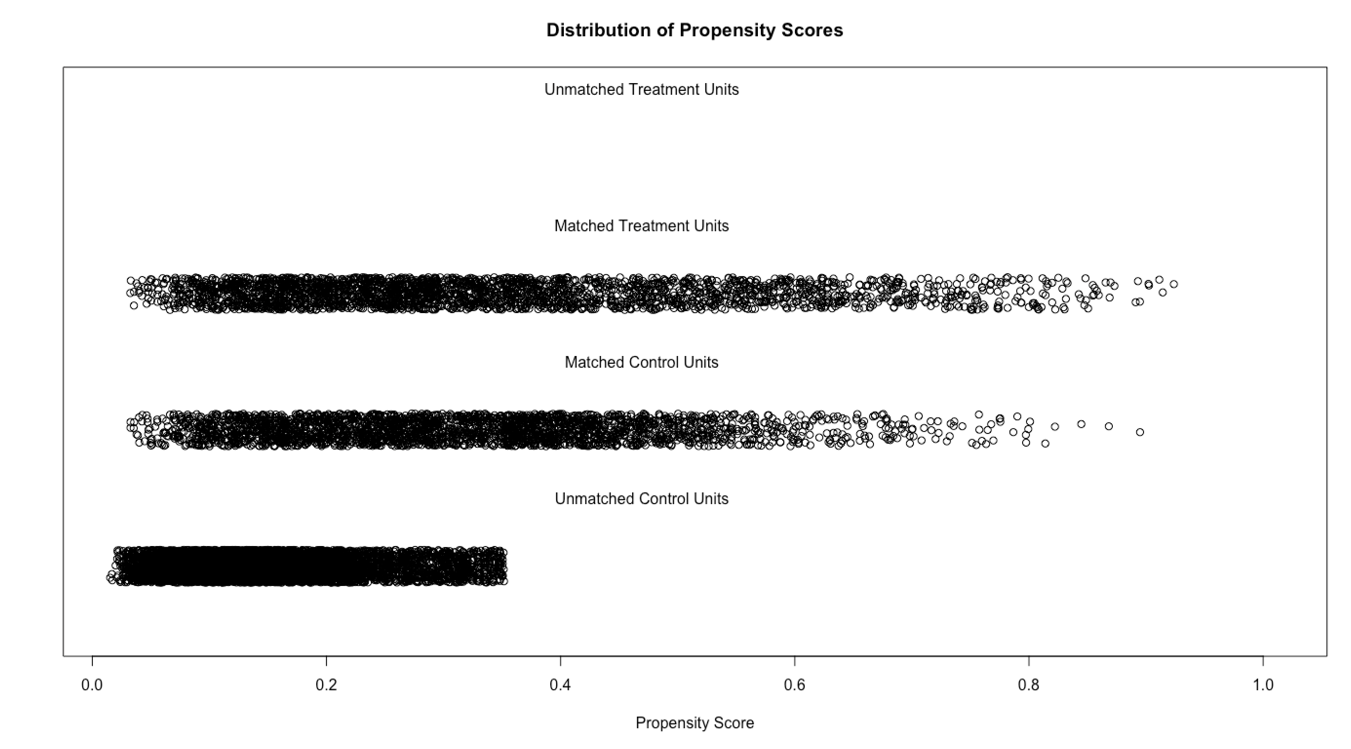


- 1. Summary of Balance for All Data


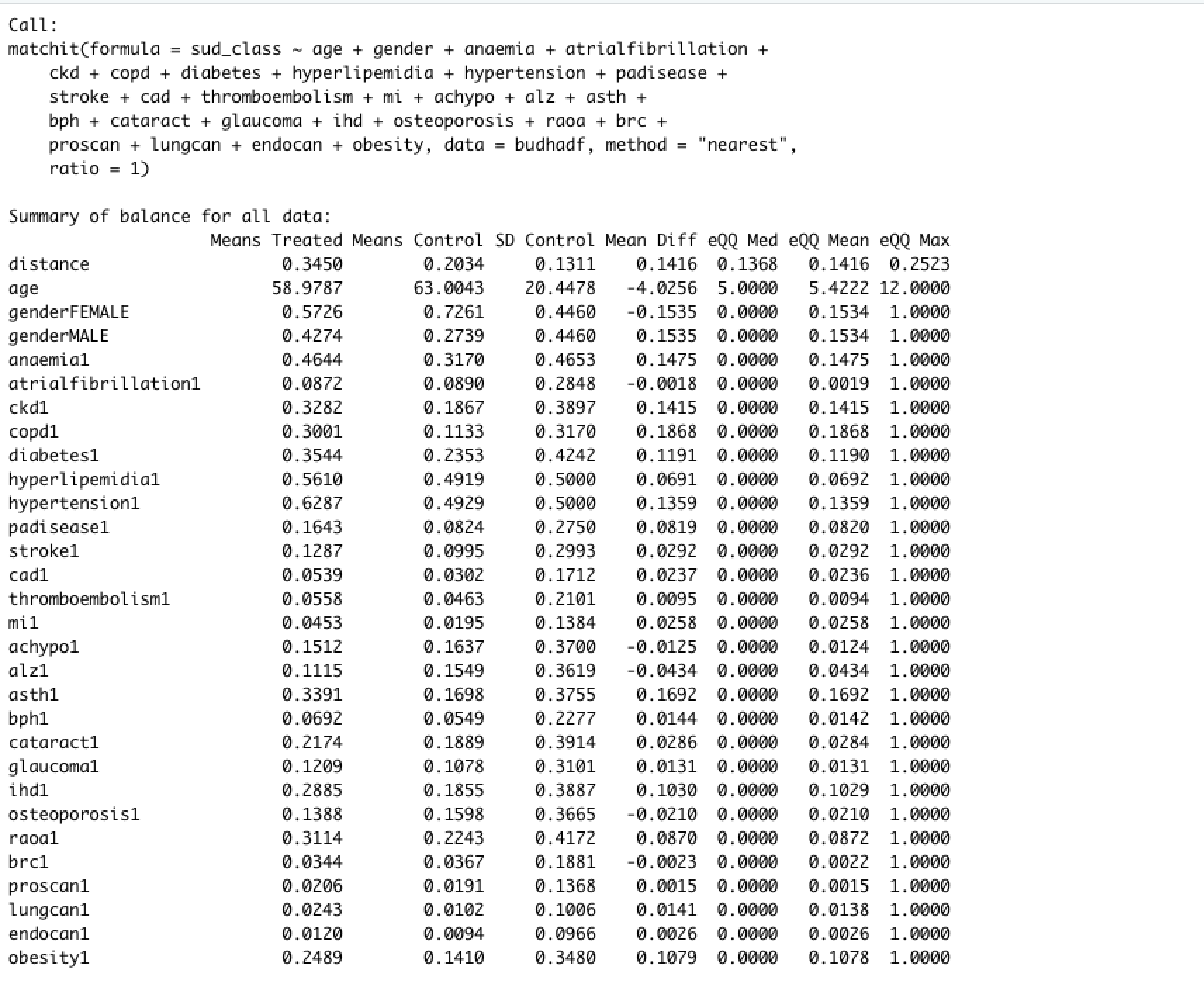


- 1. Summary of Balance for Matched Data


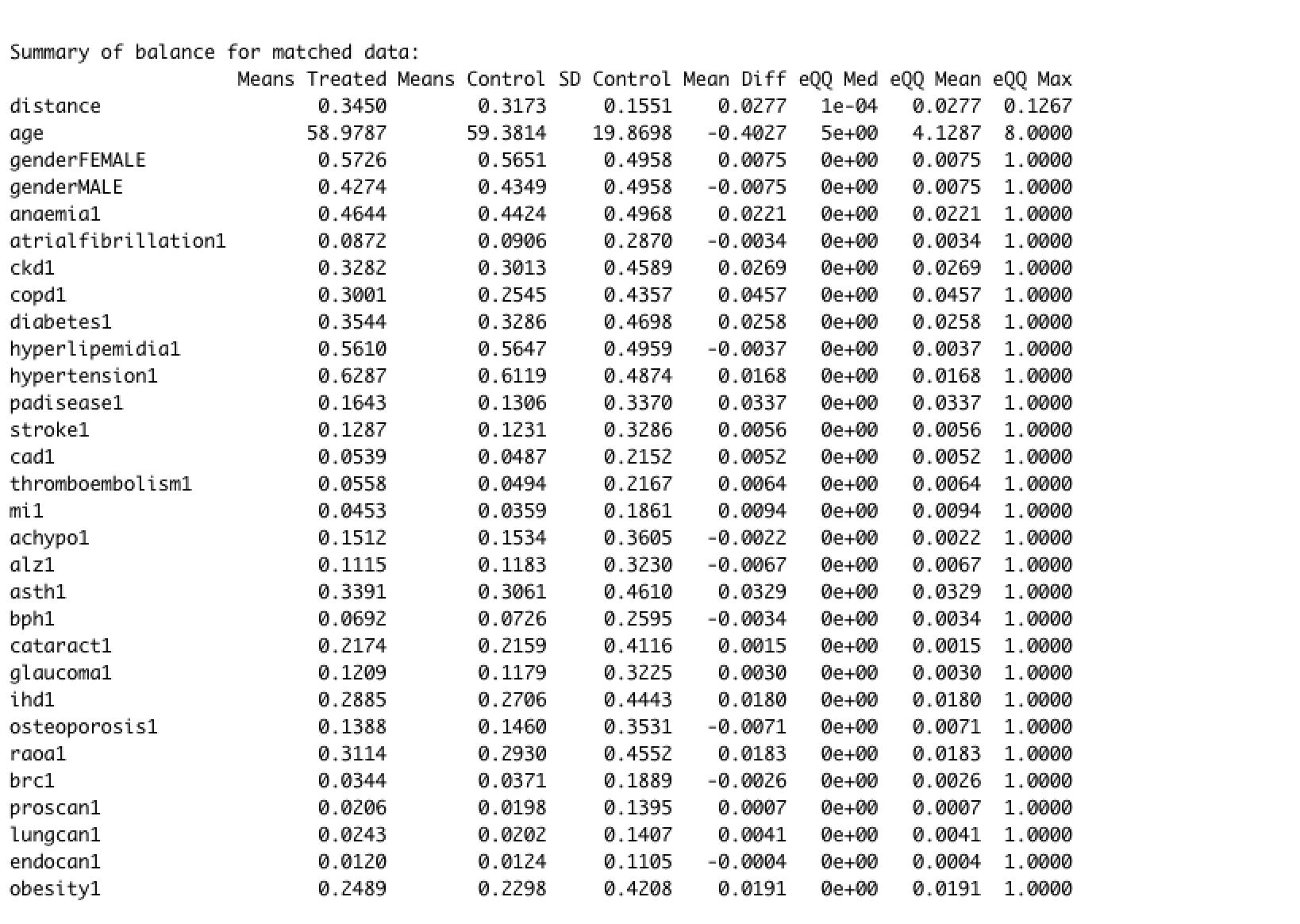


1. **Propensity Score Matching Analysis:**

**MDD-OUD (n = 424) to MDD-NOUD (n = 2 248** 🡪 **n = 424)**

- 1. Percent Balance Improvement


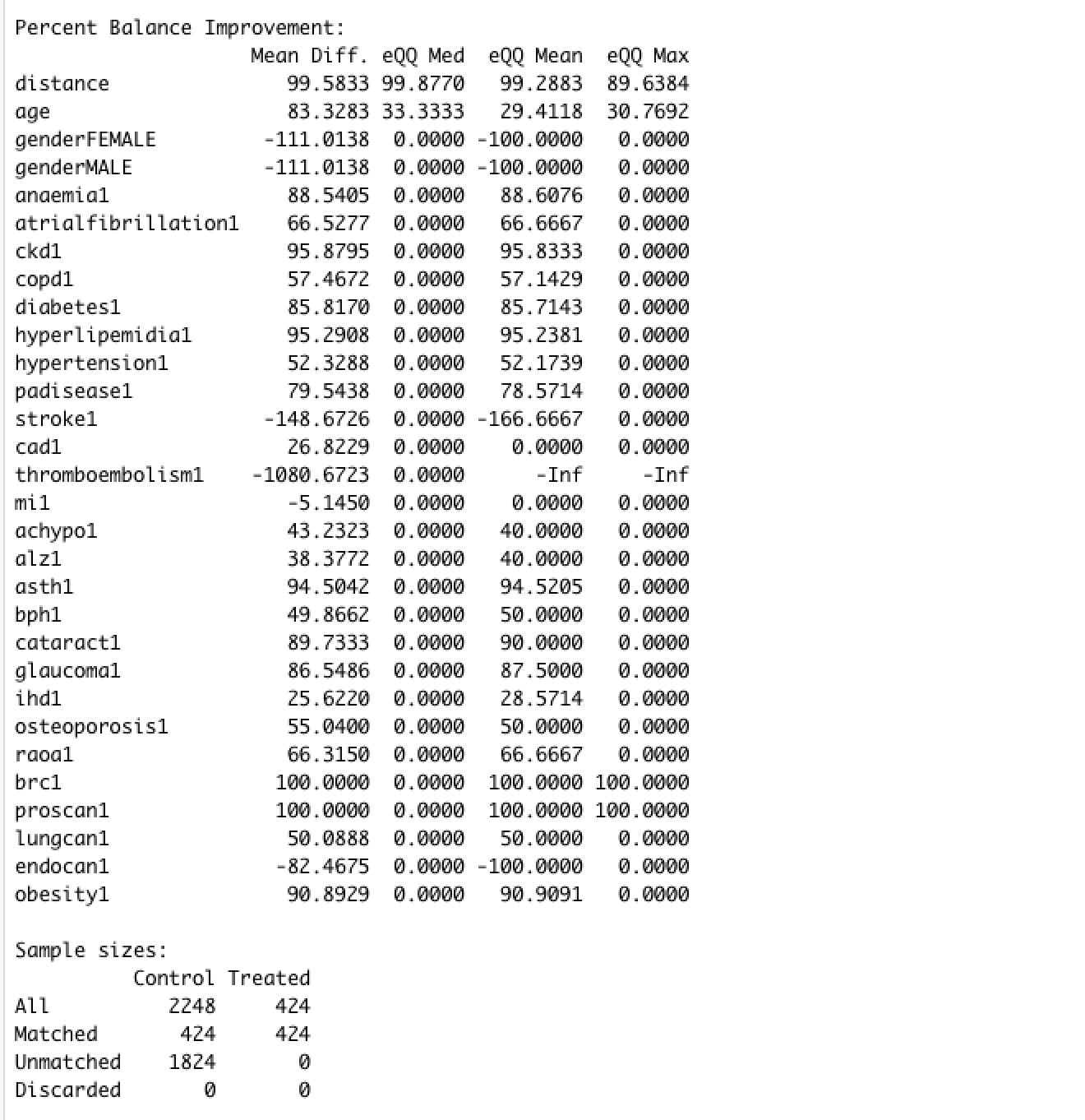


- 1. Distribution of Propensity Scores (Jitter Plot)


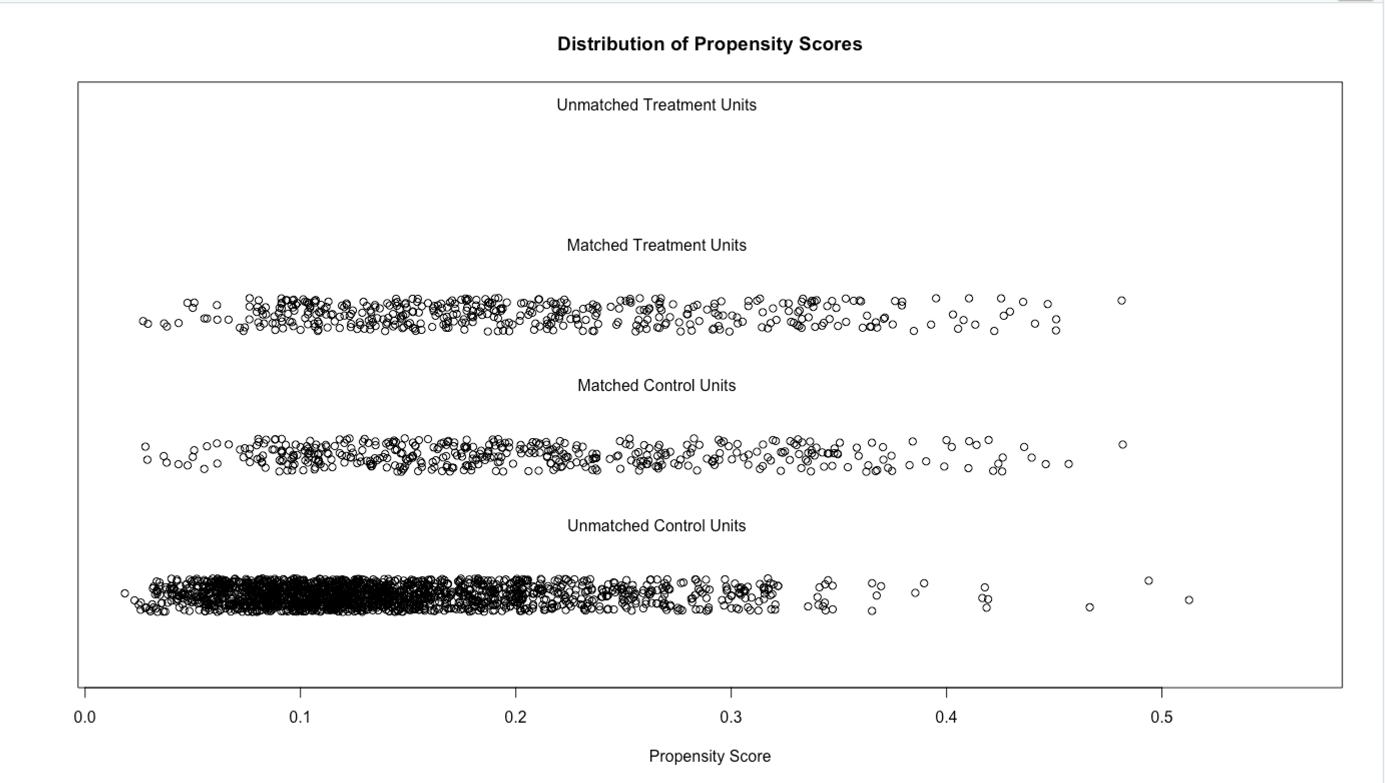


- 1. Summary of Balance for All Data


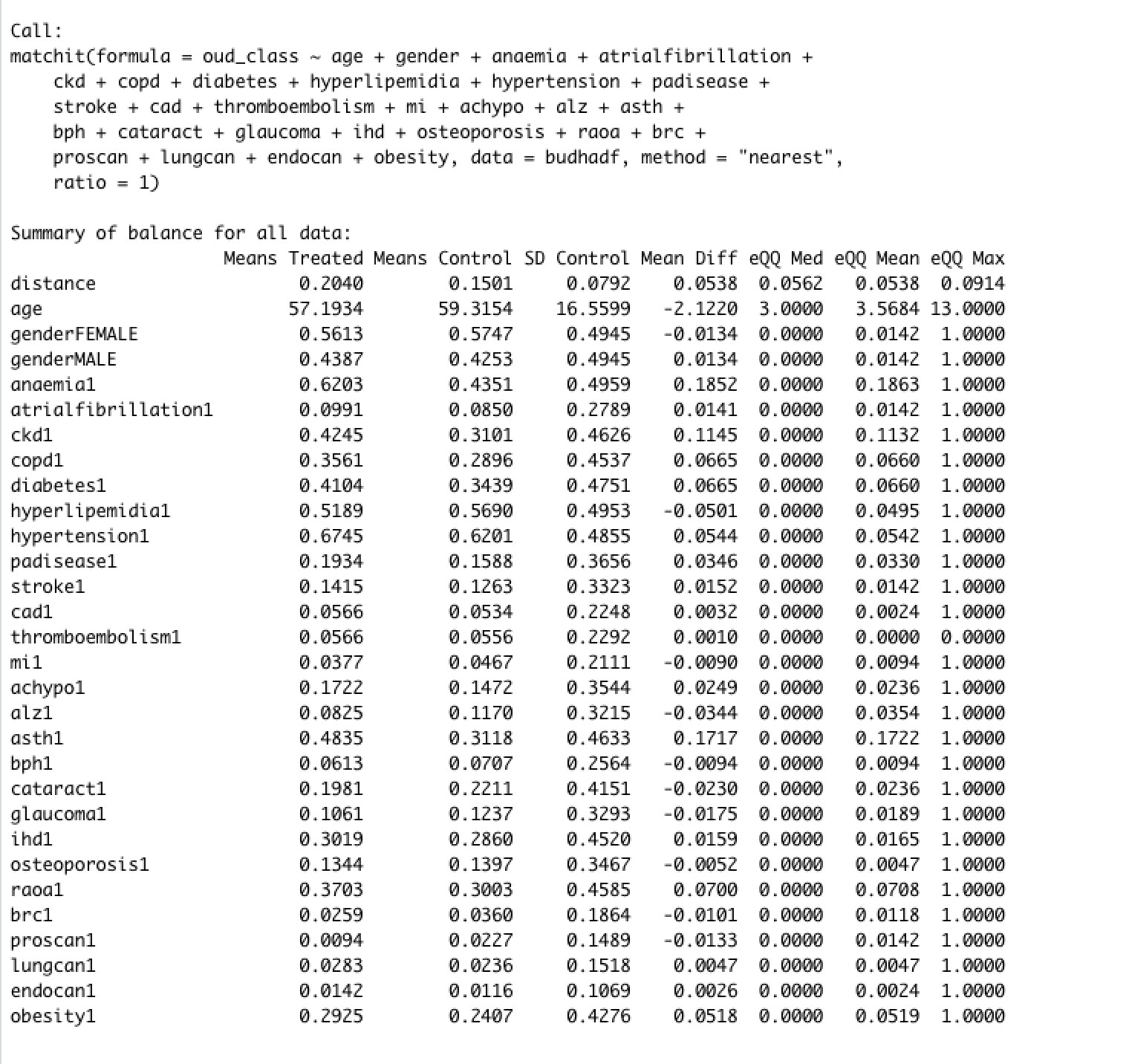


- 1. Summary of Balance for Matched Data


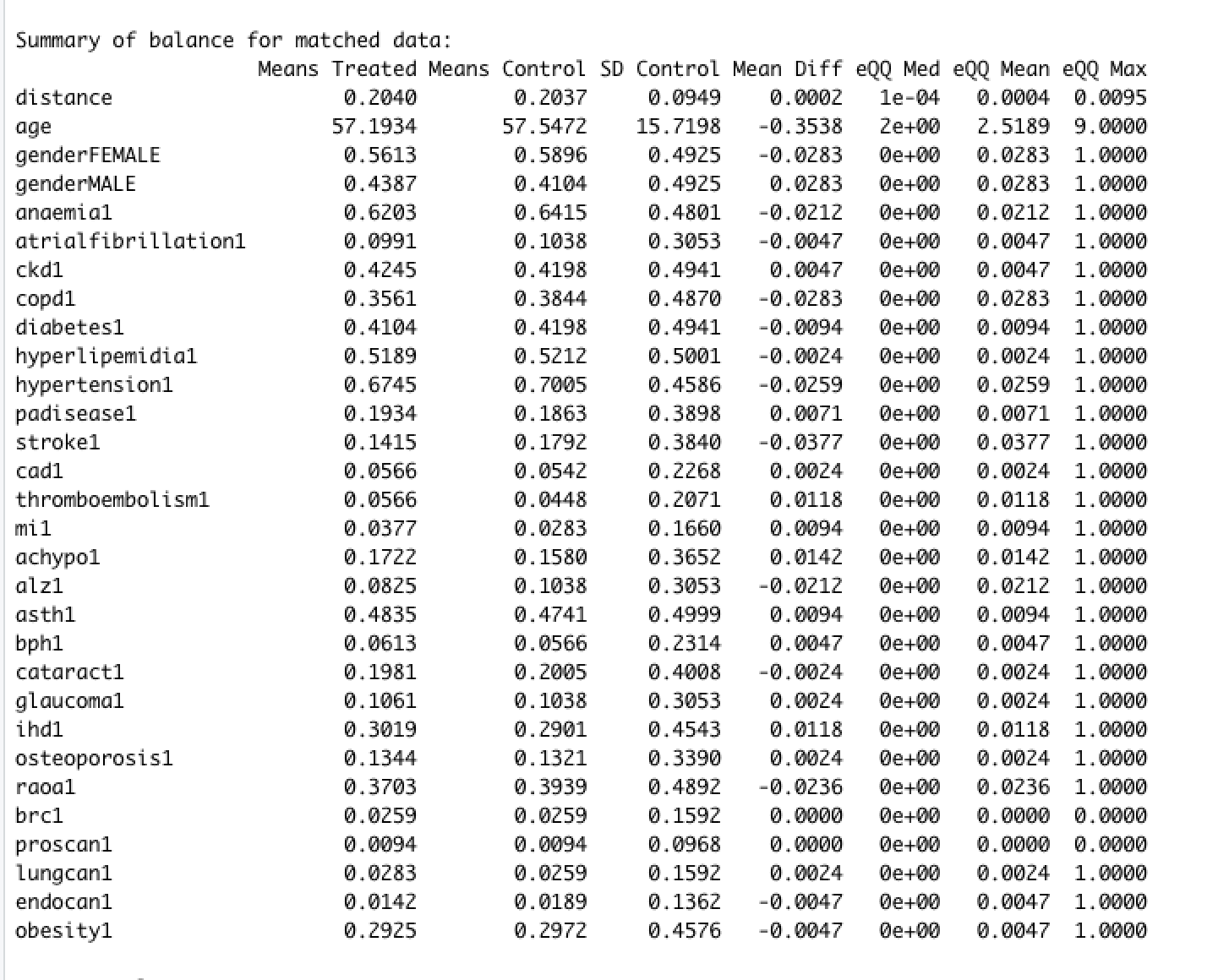

Supplement: Supplementary file 1 — Supplementary [file 41398_2021_1372_MOESM1_ESM.docx]
